# Research on the use intention of potential designers of unmanned cars based on technology acceptance model

**DOI:** 10.1371/journal.pone.0256570

**Published:** 2021-08-20

**Authors:** Tianyang Huang

**Affiliations:** School of Mechanical and Power Engineering, Guangdong Ocean University, Zhanjiang, China; Univerza v Mariboru, SLOVENIA

## Abstract

The success of unmanned car, an emerging tool of transportation with so many advantages, depends to a large extent on its user acceptability. Potential designers are both the decision makers of driverless car design and the users of driverless cars. This study aims to explore the influencing factors of the potential designers’ intention to use unmanned cars. Based on the theory of Technology Acceptance Model (TAM), this study further expanded the TAM by incorporating perceived trust, perceived enjoyment and self-efficacy, so as to explain and predict potential designers’ intention to use unmanned cars. The questionnaire is determined through theoretical literature, pre-tests, etc., and the Structural Equation Model is used to analyze the data of 202 valid survey samples to investigate the influencing factors of the willingness to use unmanned vehicles. The results show that potential designers’ intention to use unmanned cars is positively affected by perceived trust, perceived enjoyment, perceived usefulness and perceived ease of use, and perceived trust has a positive effect on perceived ease of use, self-efficacy and perceived ease of use also have a positive effect on perceived usefulness. The findings of this study can provide designers and developers of unmanned cars, policy makers and implementers with guidance in the follow-up design, policy formulation and advertising of unmanned cars.

## Introduction

There have been great concerns in the past few years on unmanned cars, one of the solutions for intelligent transportation and sustainable transportation [1]. Generally, unmanned car refers to robotic vehicle equipment without operators [2,3], also known as autonomous vehicle. It is an overturning emerging technology [2]. As artificial intelligence finds its way into every aspect of our daily life, unmanned car becomes a realistic tool and no longer stays in the concept stage. In recent years, well-known information technology companies and auto manufacturers have played an active role in the development and application of driverless cars, including China’s Baidu, Didi, Alibaba, and XPENG Motors, Dongfeng Motor. According to reports [4], as early as December 2015, Baidu’s driverless car realized fully automatic driving under mixed road conditions in cities, loops and highways for the first time in China; in 2016, Baidu’s smart car was approved by the California Vehicle Administration Approval for the road test of unmanned vehicles, which is a sign of the progress of China’s smart car technology [5]; In 2017, Baidu’s founder Robin Li took an autonomous vehicle on the Fifth Ring Road in Beijing, which caused heated discussions. At the same time, this behavior also attracted a ticket from the traffic police. At that time, China’s regulations on autonomous driving were still blank until that year. In December, Beijing, China issued a guidance document to promote actual road testing of autonomous driving, and the relevant laws and regulations for autonomous vehicles are still extremely imperfect [6]; In 2018, Baidu’s Apollo unmanned vehicle appeared at the CCTV Spring Festival Gala and started running on the Hong Kong-Zhuhai-Macao Bridge. The smart car innovation development strategy announced by China in February 2020 requires the realization of conditional automated driving smart car mass production and application in specific environments by 2025. It can be said that although the current Chinese automobile market is still dominated by traditional fuel vehicles, driven by related companies and policies, driverless vehicles and new energy vehicles are growing rapidly. However, the current acceptance of the new technology of driverless cars is poorly understood.

Unmanned cars can optimize traffic plans with the assistance of artificial intelligence technology, thus producing a plan that is more safe, efficient and less jammed with minimal manual intervention [7]. Unmanned cars may fundamentally change the way users travel [8]. A previous study stated that services such as making appointments and even reaching destinations to pick up passengers, can be provided just like providing unmanned taxi services [9]. The unmanned cars that may prevail in the near future are likely to change the existing private ownership of traditional cars, which means that users may no longer buy cars for family purposes; instead, they will call unmanned cars by making appointments when car services are needed [10]. Such a car using mode will save more land for modern private parking lots, change existing transportation methods, and conserve more gasoline energy. As pointed by Hao and Yamamoto [11], unmanned cars may reduce a lot of greenhouse gas emissions and make the environment more comfortable, achieving better environmental benefits. In the long run, with the development and popularization of the technology of unmanned cars, road congestion caused by human driving behavior can be reduced, road transportation can be more efficient [12–14], and the number of accidents caused by human errors of drivers can also get a fall [15]. All these benefits of unmanned cars require the wide application of unmanned cars in the market before becoming reality. Their application, however, is largely influenced by users’ willingness to use them [16]. As Anania et al. [17] pointed out, the success of unmanned cars is dependent on consumers’ comments on them; moreover, customers’ attitudes also affect the policies concerning unmanned cars. Therefore, it is necessary to understand the factors that affect users’ acceptance of using unmanned cars.

Early related research [18–20] explored the influence of demographic factors such as the user’s age, gender, education level, income, and residential area on the user’s willingness to use driverless cars. The results showed that users who were well-educated and young with middle income and lived in urban areas held a positive view of unmanned cars. Dedication of most of the above studies lies in the influence of demographics and some other basic information on acceptance and use of unmanned cars. Before using unmanned cars, however, users may need more trust, both the user’s trust in themselves and the trust in the unmanned car technology. In other words, the trust of users is on the basis of evaluation on cognition to their ability and belief in performance of this technology [21–23], which includes their trust in automated attributes such as the reliability and performance of unmanned cars [24,25]. Lack of trust in technical systems is the main obstacle for people to use unmanned cars [2,24]. User distrust of technical systems may lead to their not using the same, and their excessive trust may cause misuse or abuse of the same [26]. Therefore, trust, a key factor influencing human-computer interaction [27], needs to be explored in the acceptance and use of unmanned cars.

Driving a car can make people feel various pleasure and enjoyment, and that is one of the reasons why so many people buy a private car [28,29]. Obviously, manual driving which is unavailable in unmanned cars has become an interesting and important part of the driving experience [18]. Previous studies [18,30,31] showed that the higher the degree of automation, the less the hedonic benefits, and one of the important reasons lies in that the automation of unmanned cars reduces the pleasure of human driving. Unmanned cars arguably deprive the driver of the driving challenge and pleasure of skilled driving to some extent [31]. Some studies [32,33] pointed out that the perception of technology enjoyment should be considered as an important factor for users’ willingness to use technology. The perceived enjoyment is thus taken into consideration.

In addition, self-efficacy constitutes another factor that is usually overlooked in studies on the acceptance of unmanned cars. According to our previous analysis, the number of studies that incorporated self-efficacy into the use of unmanned cars has been quite limited, and there are only several studies (e.g., Lee et al. [7]) that have conducted relevant discussions. The current limited number of studies tells to a certain extent that it is necessary to carry out studies on self-efficacy’s impact on the acceptance of unmanned cars. Lee et al. [7] indicated that from a psychological perspective, users’ beliefs about their abilities are also crucial as they may think that some parts of unmanned cars are related to their abilities. Perceived self-efficacy comprises perceptual beliefs related to particular activities of users, and its measurement needs to be placed in specific situations and tasks [34,35]. For instance, in its application in health, perceived self-efficacy in health technology has a significant impact on users’ attitude towards technology use, which in turn affects their behaviors [36]. In addition, self-efficacy has also been applied to information technology fields such as computers [37]. Hohenberger, Spörrle, and Welpe [38] pointed out that self-efficacy can also be applied to studies of unmanned cars. Therefore, by combining self-efficacy with other variables, we can explore users’ willingness of using unmanned cars, understand users’ attitudes in more aspects, and improve the ability in predicting models’ acceptance of unmanned cars. Therefore, this study takes it into consideration as an important influencing factor.

As one of the most widely used theoretical models for explaining and predicting technology systems [39,40], TAM [41] has been widely applied in studies of acceptance of technology systems, such as studies of Kaur and Rampersad [2] and Zhang, et al. [42]. According to TAM, perceived ease of use and perceived usefulness are the main factors that effectively predict user behaviors. However, results of studies from different perspectives also vary individually; for example, Wu, et al. [43] and Panagiotopoulos and Dimitrakopoulos [44] suggested that the relationship between perceived ease of use, perceived usefulness and willingness of use is significant, while Buckley, et al. [45] argued that perceived ease of use has no significant effect on the willingness of use. These studies with different results showed that some aspects of TAM and their relationships remained unclear [7], and that relationship between perceived ease of use, perceived usefulness and willingness of use in some studies is not significant [45,46]. Related studies also pointed out that although TAM is a theoretical model of acceptability that was extremely widely applied, its predictive ability can still be improved by expanding the models [39,47,48]. Therefore, it is of great value to introduce TAM and conduct studies on model expansion in investigation of acceptance to unmanned cars.

In summary, unmanned cars as an emerging technology may be an effective way to solve the road safety problem in the future and has many potential advantages. For driverless cars, user acceptance is the first important content to understand. Although there have been many studies on the acceptance of autonomous vehicles, the research conducted on Chinese subjects is still insufficient. At the same time, those on the impact of self-efficacy, perceived enjoyment, and perceived trust on the acceptance of unmanned cars are still insufficient, and some study results are inconsistent, which require us to conduct further studies. Explanatory power of the TAM can have a raise by model expansion. In addition, most of the existing studies have been conducted from the public’s perspective, and no research has been conducted from the perspective of potential industrial designers. Discussing the acceptance and use of unmanned vehicles by potential industrial designers will be helpful to the design and development of unmanned vehicles. This study, based on the TAM theory and incorporating perceived enjoyment, self-efficacy and perceived trust, tries to explore the influencing factors that affect potential designers’ intention to use unmanned cars, propose and empirically apply the unmanned car acceptance models. This study intends to provide more significant factors that affect the intention to use unmanned cars, and provide more comprehensive understanding and more valuable reference for the subsequent development, studies and decision making concerning unmanned cars.

The structure of this article is as follows. The second part introduces the theoretical background and research hypothesis, and puts forward the research model, the third part introduces the research tools and other methods, the fourth part describes the research and analysis results, the fifth part discusses, and finally summarizes the research conclusions of this article.

## Theoretical background and research hypotheses

### Perceived trust

Trust is a mental state, it is the cognition and evaluation of the effectiveness of technical systems [8], and it is an important factor in the relationship between the two subjects (i.e., individuals, organizations, objects, etc.) [49]. Ward, et al. [50] pointed out that in addition to knowledge, experience and risk factors, trust factors were also related to the users’ intention to use unmanned cars. For example, in human-computer interaction, trust depends on the degree to which users control the operation of the automatic technology system [51,52], which further affects their use of automotive technology. Therefore, perceived trust can be considered as one of the key factors that drive users to use unmanned cars [8]. As Lee et al. [7] observed, studies on the relationship between perceived trust and users’ intention to use unmanned cars can make them more willing to use unmanned cars to a certain extent. Xu, Zhang’s research [53] on people’s acceptance of self-driving cars shows that trust has a significant impact on perceived ease of use and intention to use. At the same time, this relationship has also been confirmed in previous studies [54,55]. Accordingly, we proposed the following hypotheses:

**Hypothesis 1 (H1)**: Perceived trust has a positive influence on perceived ease of use.

**Hypothesis 2 (H2)**: Perceived trust has a positive influence on users’ intention to use unmanned cars.

### Perceived enjoyment

Motivation is interpreted as an internal state of an individual that drives the individual’s behavior willingness so that individual needs can be met to a certain extent [56], and hedonic motivation can be understood as the behavior of individuals’ pursuing pleasure and satisfaction [10]. Hedonic users prefer pursuing novel and complex experience and have a stronger risk tolerance capacity than others [10]. In other words, they are likely to be more willing to accept the novelty and possible risks brought about by unmanned cars [57]. It is also pointed out that hedonic motivation is one of the important predictors of the acceptance and use of unmanned cars [58]. Bay [59] also held the view that entertainment and enjoyment that users experience when using unmanned car products or services may have an impact on their intention towards the use of unmanned cars. Therefore, it is known that users who have entertainment and experience needs for unmanned cars may affect their intention to use them. Accordingly, we proposed the following hypothesis:

**Hypothesis 3 (H3)**: Perceived enjoyment has a positive influence on users’ intention to use unmanned cars.

### Self-efficacy

Bandura [60] defined self-efficacy as “Perceived self-efficacy is concerned with judgments of how well one can execute courses of action required to deal with prospective situations”, that is, the degree of a person’s belief in his ability to handle related tasks. It emphasizes the individuals’ judgment of their abilities to complete tasks, not the skill itself [60]. Self-efficacy directly affects and is one of the guiding factors of user behaviors. In previous studies related to self-efficacy, it was also pointed out that self-efficacy has a direct effect on perceived usefulness [61,62]. At the same time, Previous studies have also shown that self-efficacy has a positive influence on use intention [7]. Choi and Ji [46] regarded the concept of locus of control as perceived self-efficacy and observed that it is related to users’ intention to use a particular thing. In addition, Lee et al. [7] pointed out that self-efficacy has a positive impact on use intention in the context of unmanned cars, and is one of the important factors to predict and explain users’ acceptance and use of unmanned cars [7]. In this study, self-efficacy is understood as users’ confidence in their ability to use unmanned cars. Accordingly, we proposed the following hypotheses:

**Hypothesis 4 (H4)**: Self-efficacy has a positive influence on perceived usefulness.

**Hypothesis 5 (H5)**: Self-efficacy has a positive influence on users’ intention to use unmanned cars.

### Technology Acceptance Model (TAM)

The TAM proposed by Davis [41] is considered one of the most widely applied models in information technology systems. It can be employed to predict and understand users’ acceptance of technology systems. For example, Ma and Chan [63] conducted research on key factors affecting the use of smartphones by the elderly based on theories such as Technology Acceptance Model (TAM), while Xu and Zhang [53] and Buckley & Kaye [45] conducted research on the basis of TAM Car driving acceptance study. Perceived usefulness and perceived ease of use, as important factors in the theory, can predict and explain users’ acceptance of technology systems, the latter has a positive impact on the former, and the two have a positive impact on willingness to use a technology. For example, Yousafzai and Foxall [64] also pointed out that perceived ease of use has a direct and positive impact on perceived usefulness. At the same time, the research of Zhang and Tao [65] also confirmed a relationship. In the past, there have also been studies that make TAM a theoretical basis in the investigation of acceptance of unmanned cars. However, in such studies in the past, the relationship between the factors in the original TAM was different due to the difference in focuses of such studies. As a consequence, the study results varied as well. For example, Lee et al. [7] proposed that perceived ease of use has an impact on perceived usefulness, perceived usefulness has an impact on use intention, and perceived ease of use has no impact on use intention. The study of Buckley et al. [45] also showed that perceived ease of use has no significant impact on use intention. By contrast, some study results suggest that both perceived ease of use and perceived usefulness have a direct and positive impact on use intention [43,44]. The above studies showed that, in addition to the indirect impact on use intention, perceived ease of use may also have direct and positive impact. Furthermore, great benefits of unmanned cars may also reinforce users’ willingness to use them [46,53]. Although some previous study results showed that the relationship among core factors of TAM is different from the TAM models, However, TAM as the most widely applied technology acceptance model, keeps the basic assumptions between its factors stable. In addition, in the course of study on technology acceptance, it is particularly useful in the early stages of technology development to take behavioral willingness, rather than actual use, as a dependent variable [46,66–68]. Therefore, we make users’ willingness to use unmanned cars as the outcome variable. In summary, we proposed the following hypotheses:

**Hypothesis 6 (H6)**: Perceived ease of use has a positive impact on perceived usefulness.

**Hypothesis 7 (H7)**: Perceived ease of use has a positive impact on users’ intention to use unmanned cars.

**Hypothesis 8 (H8)**: Perceived usefulness has a positive impact on users’ intention to use unmanned cars.

**Fig 1:**
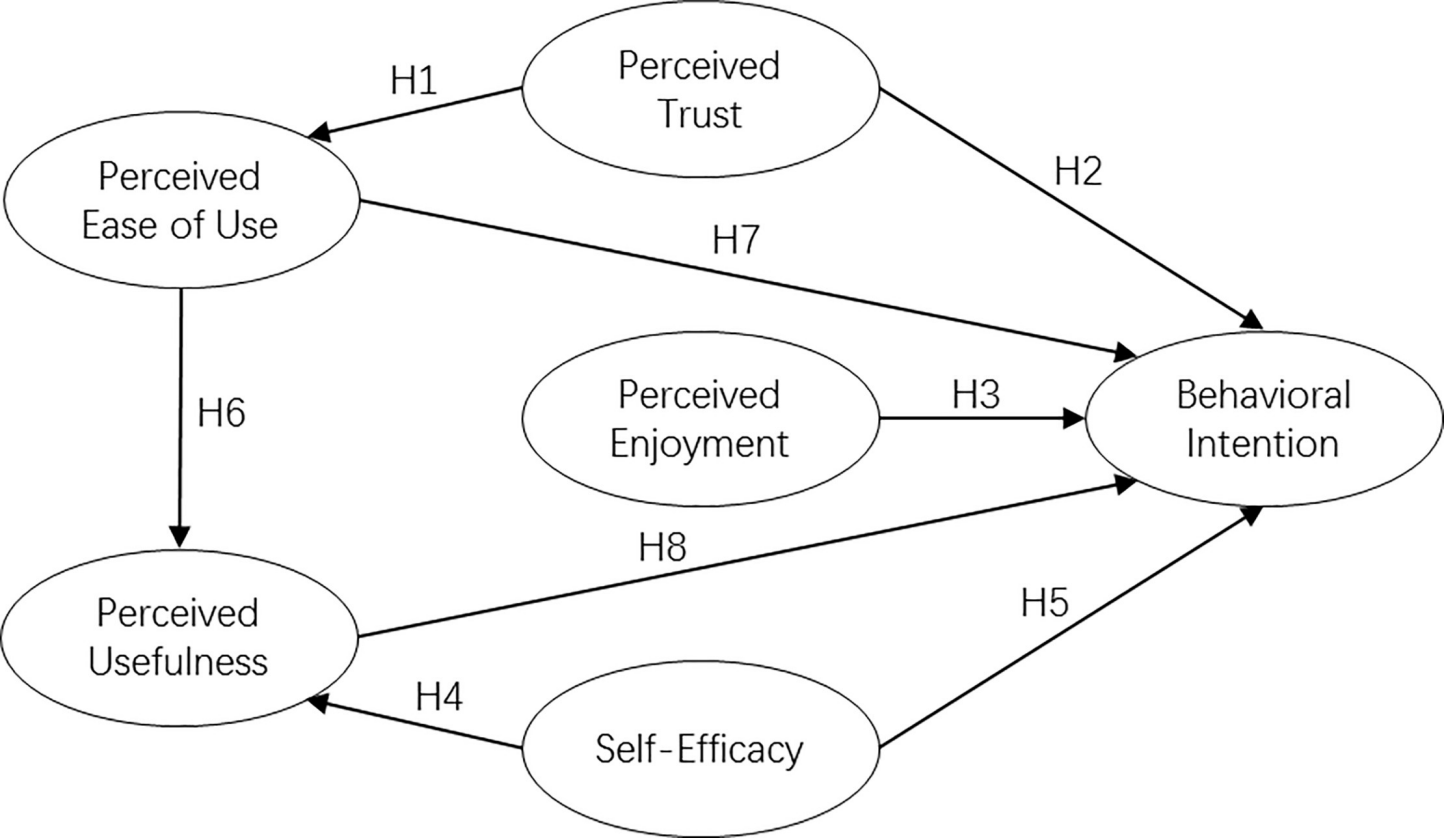
Research structure.

In summary, the structure of this study is proposed, as shown in Fig 1.

## Methods

### Subjects

Our research objects are potential industrial designers. Potential designers are not only the future decision-makers of driverless car design, but also the potential users of driverless cars. Therefore, this study collected relevant data from students of three grades majoring in industrial design at a university in Guangdong Province, China, by convenience sampling method. We consider that these young research objects will move towards designer positions such as appearance design, car structure design, and design management related to driverless cars in the next 1–3 years. They are potential designers and have a certain degree of representativeness. Those groups that do not have design knowledge may be a broader public group, and professional designers with rich experience may have a certain impact on the understanding of research topics because of their professional experience. Therefore, the freshmen who just entered a semester did not start to design related courses and professional designers with practical work experience were not included in the scope of this research, and they did not participate in this research. The ethics application for this study was approved by the Academic Committee of Guangdong Ocean University. At the same time, all participants verbal agreed to participate in the study under the recruitment and witness of the researchers and the head teacher, and took the initiative to fill in and submit the research questionnaire.

### Research tools

This study verified the hypotheses by using questionnaire scales to collect data. The questionnaire items are based on this research theme, and effectiveness of content therein is validated through adaptation of theoretical literature and expert evaluation. The pre-test of 27 questionnaires showed that all of the questionnaire items can be well understood. The Cronbach’s alpha value of each variable was higher than 0.7, indicating that the questionnaire constructs meet the reliability requirements. On this basis, the formal questionnaire scale covering 6 dimensions in the study framework and 21 items was established. Of these dimensions, the measurement indicators of perceived enjoyment were adapted from Madigan et al. [58], the measurement indicators of self-efficacy were adapted from Compeau and Higgins [69], the measurement indicators of perceived trust were adapted from Choi and Ji [46], and the measurement indicators of perceived usefulness, perceived ease of use, and use intention were adapted from Davis [41]. In addition, the questionnaire also designed questions of demographics, including gender, age, driving license holding conditions, Autonomous vehicles awareness, driving experience and other basic information. See the support information for the complete questionnaire. All participants were asked to answer the questionnaire items on a Likert 7-point scale, ranging from 1 (strongly disagree) to 7 (strongly agree), while 3 is (neutral).

### Data collection and analysis

Our questionnaire was based on online survey and data collection, and the online questionnaire survey platform applied is called Questionnaire Star (https://www.wjx.cn/), a professional online questionnaire survey platform in China. The questionnaire was made anonymously and, to prevent repeated participation, each IP was set to participate in answer once only. The time of the questionnaire survey was from February 8, 2021 to February 27, 2021. a total of 216 questionnaires were collected and 202 were deemed eventually valid the then time after deletion of 14 due to the answer time is too short and the answers are consistent, answer choosing on a regular basis. We verified hypotheses in the study by Partial Least Squares taking into account its advantage in multivariate analysis [70]. In order to obtain a more stable estimated coefficient, repeated sampling [71] of 5,000 times was used to calculate the significance of the path coefficient.

## Results

### Sample description

The basic information of the sample data is shown in Table 1. The average age of 202 participants is 21 years (SD = 1.142). There are 110 (54.5%) males and 92 (45.5%) females, of whom 68 (33.6%) are second-grade students, 67 (33.2%) are third-grade students, and 67 (33.2%) are forth-grade students. More than half of the participants have obtained a small car license, that is, a private car driver’s license, not a truck driver’s license (n = 106, 52.5%) and 89 (44.1%) have driving experience. Most of the participants (n = 188, 93.1%) have heard of AVs, and only a few (n = 14, 6.9%) have not.

**Table 1:** Statistical description (*N* = 202).

Items	Option	Number	Percentage (%)
Gender	Male	110	54.5
	Female	92	45.5
Grade	Second-grade	68	33.6
	Third-grade	67	33.2
	Forth-grade	67	33.2
Small car license	Have	106	52.5
	No	96	47.5
Driving experience	Have	89	44.1
	No	113	55.9
Heard of AVs	Have	188	93.1
	No	14	6.9

Note: 1. The object of this research is a potential industrial designer, that is, industrial design students in grades 2–4, and the valid sample is 202; 2. The average age of the participants in the valid questionnaire is 21 years old.

### Reliability and validity of the questionnaire

Using IBM SPSS 25.0 to perform exploratory factor analysis on all measurement items, the KMO value was 0.901, the Bartlett sphere test chi-square value was 1254.663, P<0.001, and the factor loads after the maximum variation method were rotated were greater than 0.5[72], and It has a good one-to-one correspondence with its corresponding variables. Considering the questionnaire method used in this study, the main variables are all from the same interviewee. At the same time, in order to prevent potential common method deviations caused by potential factors such as participants’ emotional state and motivation to participate [73], we answered anonymously, concise description of the topic and other program control methods to reduce the common method deviation. In addition, we also use the Harman single factor test method to calculate the maximum variance explanation rate of a single factor in the model to evaluate the impact of common method bias [73,74]. The results show that the degree of variance explained by a single factor maximizing is 40.662%, which does not exceed the 50% standard [73,74], which indicates that the common method bias has no significant impact on this study.

Confirmatory factor analysis was applied to test and measure the reliability and validity of the variables, and the results are shown in Tables 2 and 3. To know the stability and reliability of the variables, this study applied Cronbach’s alpha reliability coefficient value and composite reliability. The results showed that the Cronbach’s alpha value and composite reliability coefficient of all dimensions exceeded the 0.7 standard recommended by Fornell and Larcker [75], indicating that all dimensions performed well in reliability. Average Variance Extracted values of all dimensions were higher than 0.5, indicating that the measurement also performed well in convergence validity [76,77]. According to Fornell-Larcker criterion, the discriminative validity can be evaluated by comparing the square root value of AVE of each dimension with the correlation coefficients of other dimensions [75,76]. According to Table 3, square root value of AVE of each dimension is higher than its correlation coefficient with other dimensions, which indicated that the measured variables performed well in discriminative validity.

**Table 2:** Mean, standard deviation, reliability and validity of variables.

Constructs	Item	Indicator reliability	Mean	Std. Dev.	Cronbach’s Alpha	CR	AVE
PEOU	PEOU1	0.766	4.762	0.969	0.725	0.844	0.643
	PEOU 2	0.795					
	PEOU 3	0.843					
PU	PU1	0.824	4.752	0.949	0.802	0.869	0.625
	PU2	0.770					
	PU3	0.771					
	PU4	0.796					
PT	PT1	0.840	4.455	1.028	0.790	0.877	0.704
	PT2	0.842					
	PT3	0.836					
PE	PE1	0.835	4.818	0.953	0.751	0.858	0.668
	PE2	0.818					
	PE3	0.799					
SE	SE1	0.711	4.792	1.040	0.852	0.894	0.630
	SE2	0.837					
	SE3	0.717					
	SE4	0.818					
	SE5	0.872					
BI	BI1	0.860	4.764	1.108	0.826	0.890	0.742
	BI2	0.843					
	BI3	0.880					

**Table 3:** Correlation coefficient between various dimensions.

	Behavioral Intention	Perceived Ease of Use	Perceived Enjoyment	Perceived Trust	Perceived Usefulness	Self-Efficacy
Behavioral Intention	0.861					
Perceived Ease of Use	0.524	0.802				
Perceived Enjoyment	0.596	0.450	0.817			
Perceived Trust	0.618	0.390	0.491	0.839		
Perceived Usefulness	0.682	0.522	0.652	0.545	0.790	
Self-Efficacy	0.567	0.542	0.647	0.609	0.544	0.794

Note: The diagonal is the square root value of the average variance extraction amount.

### Hypotheses testing of the study

**Fig 2:**
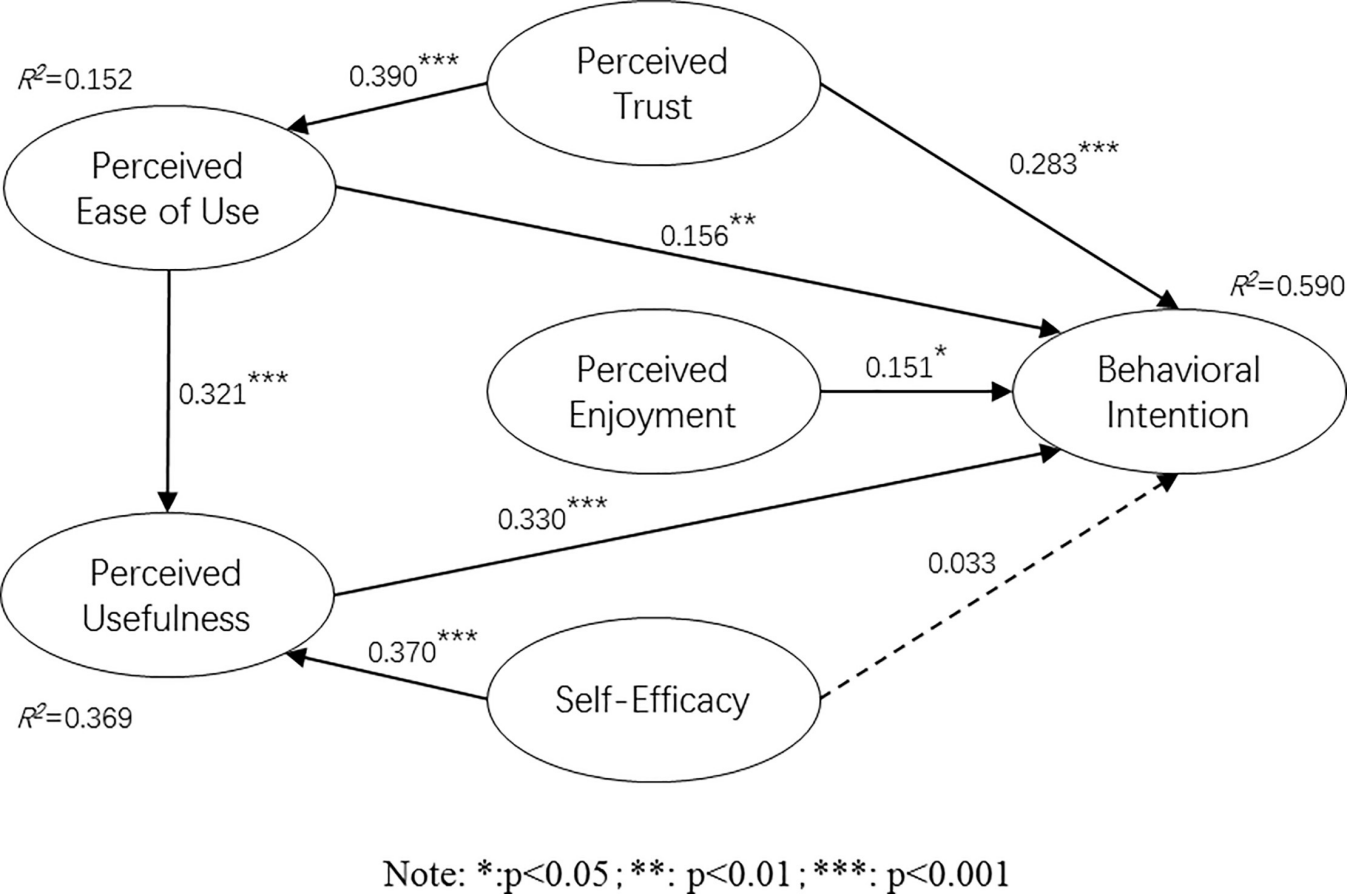
Model verification results.

The model test results are shown in Fig 2. As seen, the R2 value of the entire model is 0.590, between the medium explanatory power 0.33–0.67 standard recommended by Henseler and Ringle [78],which indicates that the entire model explains 59% of the variance in willingness to use unmanned vehicles, and that the perceived ease of use, perceived usefulness, perceived trust, perceived enjoyment have strong explanatory power for the willingness to use unmanned cars. The R2 value of perceived usefulness is 0.369, indicating that perceived ease of use and self-efficacy have moderate explanatory power for perceived usefulness. The R2 value of perceived ease of use is 0.152, which satisfies Falk and Miller’s [79] suggestion that R2 is higher than 10% with independent explanatory power. Therefore, the R2 in this study has reached the standard. The Goodness of fit criterion is regarded as the fitness index of PLS [80], which ranges from 0.00 to 1.00, and the GOF values of large, medium and small effects are 0.10, 0.25, 0.36 [71,81]. The fitness index GoF value of this research model is 0.498, which is a large effect size exceeding the cutoff value of 0.36 [71], indicating that the sample data of this research has a high degree of adaptation to the model.

The results show that perceived trust has a significant impact on perceived ease of use (ß = 0.390, p < 0.001) and on use intention (ß = 0.283, p < 0.001), so H1 and H2 are supported. The influence coefficient of perceived enjoyment on use intention is 0.151 (p < 0.05), which indicates that perceived enjoyment has a significant positive impact on the intention to use unmanned cars, so the H3 is verified. The results also show that self-efficacy has a positive impact on perceived usefulness (ß = 0.370, p < 0.001) at a less significant level (ß = 0.033, p > 0.05), so H5 is not verified and H4 are supported; perceived ease of use has a significant positive impact on both perceived usefulness (ß = 0.321, p < 0.001) and on use intention (ß = 0.156, p <0.01), so H6 and H7 are supported; and the perceived usefulness also has a positive effect on use intention (ß = 0.330, p < 0.001), so H8 is supported.

In addition, we assume that perceived trust indirectly affects intention to use through perceived ease of use, and self-efficacy indirectly affects intention to use through perceived usefulness. Since the Bootstrap method is relatively unlimited in use, it is particularly suitable for PLS-SEM, and has a higher statistical verification power than the Sobel test [82]. The model uses Bootstrap to verify multiple mediation effects, and the results are shown in Table 4. At the same time, use the rule of thumb of VAF> 80% (Full Mediation); VAF ≤ 80% (Partial mediation) and VAF <20% (No mediation) recommended by Hair, Hult, Ringle and Sarstedt [82] to determine mediation. It can be seen from Table 4 that perceived usefulness plays a role as an intermediary between perceived ease of use and intention to use. Its indirect effect is 0.106, t value is 2.703, p<0.01, and its VAF value is 40.46%, which indicates that perception Usefulness has a partial intermediary effect. Perceived usefulness also plays an intermediary role in the relationship between self-efficacy and intention to use. Its indirect effect value is 0.122, t value is 3.499, p<0.001, and its VAF value is 78.71%, which means that perceived usefulness has a partial intermediary effect.

**Table 4:** Mediation effect test.

Independent variable	Mediators	Dependent variable	Direct effect	Indirect effect	Total indirect effect	VAF
Perceived Trust	Perceived Ease of Use	Behavioral Intention	0.283***(4.349)	0.061**(2.618)	0.344	17.73%
Perceived Ease of Use	Perceived Usefulness		0.156**(2.940)	0.106**(2.703)	0.262	40.46%
Self-Efficacy	Perceived Usefulness		0.033(0.420)	0.122***(3.499)	0.155	78.71%

Note

**, p<0.01

***, p<0.001; () The value inside is the t value; VAF, variance accounted for.

## Discussion

This study was on the basis of and expanded TAM theory by incorporating another three dimensions–perceived trust, perceived enjoyment and self-efficacy, and constructed and demonstrated the acceptance model of unmanned cars. The results show that the perceived trust, perceived enjoyment, self-efficacy, perceived usefulness and perceived ease of use are applicable to explain and predict potential designers’ intention to use unmanned cars. Therefore, the results of the study are of guiding significance for the design of unmanned vehicles and relevant policy formulation. In the future research on driverless cars, we should integrate the research foundation of the predecessors and the results of this research, through the market promoters, designers and decision makers of driverless cars, from product design, product promotion and user develop design and planning at equal levels. For example, at the product level, designers improve the usability and ease of use of driverless cars from aspects such as ergonomics, operability, experience, and human-computer interaction, and enrich their entertainment. At the user level, through the relevant market promoters of driverless cars, increase the promotion of product usability and other features, so as to obtain users’ perceptions of the usefulness, ease of use and entertainment of driverless cars, and ultimately enhance users’ the goodwill of driverless cars and enhance users’ trust in driverless car products.

This study also found that perceived trust has a significant impact on both perceived ease of use and use intention. Panagiotopoulos and Dimitrakopoulos [44] and Liu, Xu & Zhao [83] confirmed the positive impact of perceived trust on the use intention. Only if users trust AVs as an emerging technology, will they have a better understanding of its ease of use, and develop further intention to use them. However, Kaur and Rampersad [2] did not suggest a significant effect of perceived trust, which may be due to differences in study objects or models. The positive effect of perceived trust on perceived ease of use and use intention indicates that relevant organizations should lay more efforts on higher credibility of unmanned cars. In addition, an emerging technology in the early development stage may not be trusted due to users’ unfamiliarity and ignorance of it, which infers that for further development of unmanned cars, manufacturers and related organizations should increase publicity efforts, enhance user trust of and make them know more about unmanned cars by making policies, advertising and inviting users to experience this novel technology, so as to affects users’ perception of the ease of use of unmanned cars and ultimately enhance their use intention.

Results of our study show that perceived enjoyment directly affects users’ willingness to use unmanned cars, consistent with the results of Hegner et al. [8] and Keszey [10], which suggests that perceived enjoyment is related to users’ technical use. For this reason, we should attach importance to this correlation to play the hedonic effect of unmanned cars. The young people in this study may be more concerned about the entertainment of technology, and they may try to enrich their spiritual world and emotional experience through the entertainment of technology. This also makes the results of this study show that perceived entertainment has a positive impact on willingness to use. It has been pointed out in the past studies that hedonic motivation plays an important role in the motivations of using cars [84]. According to our study results, entertainment features should be added to the subsequent AVs to enrich the users’ driving experience. However, it has also been pointed out in some studies that, as automation grows stronger, the role of hedonic benefits may decline [18,31]. This is because automation deprives users of the opportunity to obtain pleasure from their manual driving; in addition, as the degree of automation increases, the psychological distance between users and cars is also being farther [31]. Taking this into account, R&D staff and designers of unmanned cars should fully consider users’ need for entertainment, as well as its relationship with the use intention, trying to maintain the entertainment experience of drivers in unmanned cars while achieving automation, thus meeting users’ pursuit of entertainment experience.

The results of this study show that self-efficacy does not directly affect users’ willingness to use unmanned cars, but indirectly by affecting their perceived usefulness. However, the research of Lee et al. [7] shows the direct effect of self-efficacy on perceived ease of use and willingness to use. They argued that self-efficacy is a prerequisite for perceived ease of use. The difference between our study results with theirs may be a result of differences in user groups that are studied. This study found that self-efficacy has no direct effect on use intention, and the reason may be that participants believe the ability to operate AVs may not be so important for intention to use them as they are driverless, these potential designers may be more concerned about the fully automated experience of the product. Self-efficacy has no direct effect on the users’ intention to use unmanned cars, but our study implicates that the positive impact of self-efficacy on perceived usefulness may also reflect to some extent the relationship between the users’ ability and technical usefulness. In other words, the users’ perceived usefulness of unmanned cars may be limited by the capability of users. On this ground, requirements on user capability for unmanned cars can be weakened in the follow-up advertising of unmanned cars, so as to break it to the existing concepts of participants, that is, technical products may be useful only if their competent to use them. All-round publicity such as the ease of use of AVs and user guides may be applied to reduce users’ concerns over their ability to operate products. Of course, users’ operational capabilities can be improved to enhance their awareness of and willingness to use unmanned cars.

This study also explored the impact of perceived usefulness and perceived ease of use on use intention, and found the positive impact of the former two on the latter. The decisive effect of perceived ease of use on use intention was also confirmed in previous studies [15,44]. The results also suggest that perceived ease of use has a positive impact on perceived usefulness, which was also validated by Zhang et al. [65]. Considering the positive role of perceived ease of use in constructing perceived usefulness of unmanned cars and users’ willingness to use, the performance of perceived usefulness should be overemphasized. For example, users’ perceived usefulness can be enhanced in subsequent design of unmanned cars through design of human-computer interfaces. Users’ perception of usefulness, of course, can also be improved through the intelligent simple operation and control of unmanned cars, as well as the benefits of unmanned cars, so as to highlight the unique advantages of unmanned cars and then strengthen users’ willingness to use them. If the usefulness and other advantages of unmanned cars are not highlighted, users may not be willing to use them. As Pakusch et al. [13] noted that, users pay little attention to the advantages of unmanned cars, leading to a result that those who already own private cars will not try unmanned cars.

The study has offered more understanding over influencing factors of intention to use unmanned cars, and is of reference value for subsequent studies, design and advertising of unmanned cars. The study incorporates self-efficacy and other less discussed dimensions in unmanned cars into the TAM, which further expands the TAM and its application fields. In addition, this study distinguishes itself from previous studies targeted to the public by making potential industrial designers as its subjects, so as to enrich studies on users’ acceptance of unmanned cars. However, our study also has some limitations. First of all, our study subjects did not actually drive or experience unmanned, which may have certain impact on the results. In the future we can strengthen users’ understanding and trust in unmanned cars by inviting them to drive and test, and the subsequent study on acceptance and use intention may produce some interesting findings. Self-efficacy can also be fortified through experience, demonstration, guidance, etc., which further affects user behaviors. From this point this study is also limited by its nature of being a cross-sectional study. In the future, longitudinal studies may be tried to explore impact of self-efficacy, perceived trust, etc. that may vary with time and experience on users’ technology use behaviors. At the same time, the research object comes from Guangdong Province, China, which limits the general applicability of the research results to other countries or regions to a certain extent. Second, the heterogeneity of the respondents (such as gender, experience) may limit the power and generalizability of this study. Finally, although convenience sampling is widely used as an effective method, this method may have certain limitations on the results of this research. In the follow-up, we can try to use diversified sampling methods for topic research and expand potential designers to Enrich research objects.

## Conclusions

In order to make unmanned cars to be accepted by more, it is important to understand the factors affecting intention to use unmanned cars. Our study found that perceived trust, perceived enjoyment, perceived usefulness and perceived ease of use have a significant impact on use intention, that Self-efficacy and perceived ease of use have a positive impact on perceived usefulness, and that perceived trust has a significant impact on perceived ease of use. The results of this study make people know more about such a group who are both potential industrial designers and potential users of AVs, which is of reference value to improve acceptance and optimize design of AVs.

## Supporting information

S1 Fig Research structure. (TIF)

S2 Fig Model verification results. (TIF)

S1 File Questionnaire. (DOCX)
