# Supplementary figures and images for: Research on the use intention of potential designers of unmanned cars based on technology acceptance model
Source: PLoS One. 2021 Aug 20;16(8):e0256570. doi: 10.1371/journal.pone.0256570 (PMC8378682; doi:10.1371/journal.pone.0256570)

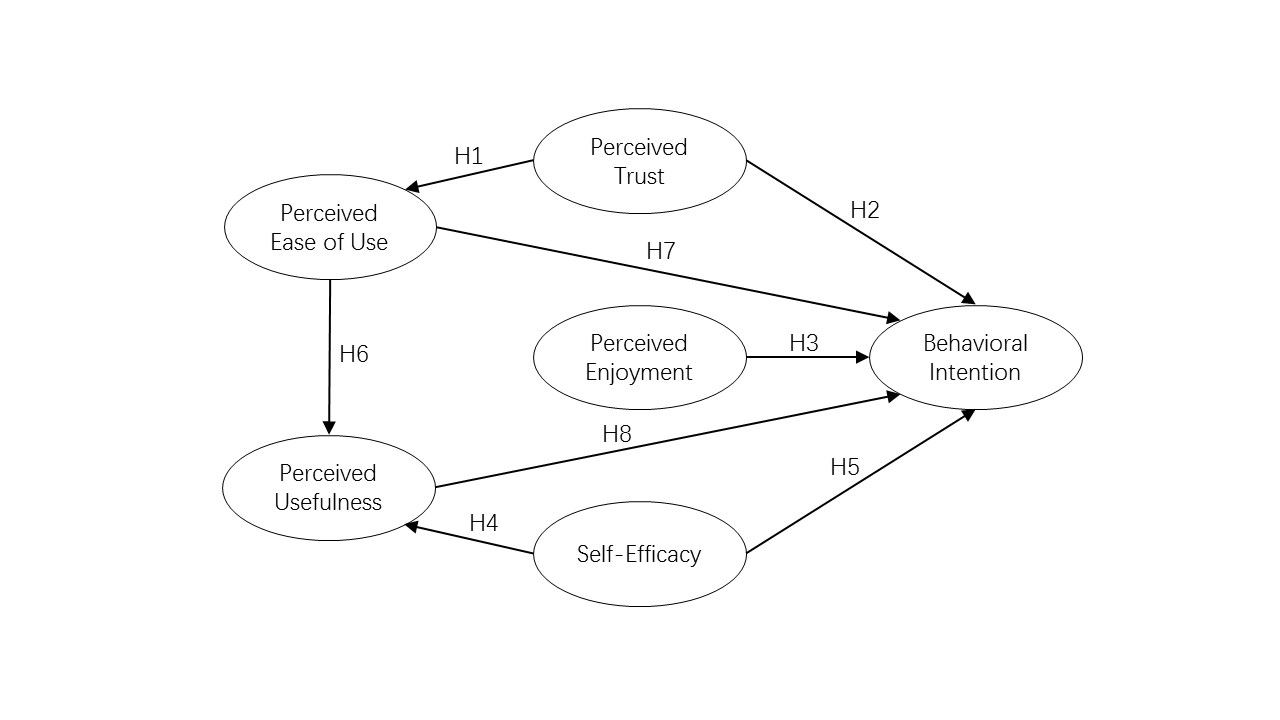

Supplement: S1 Fig — (TIF) [file pone.0256570.s001.tif]

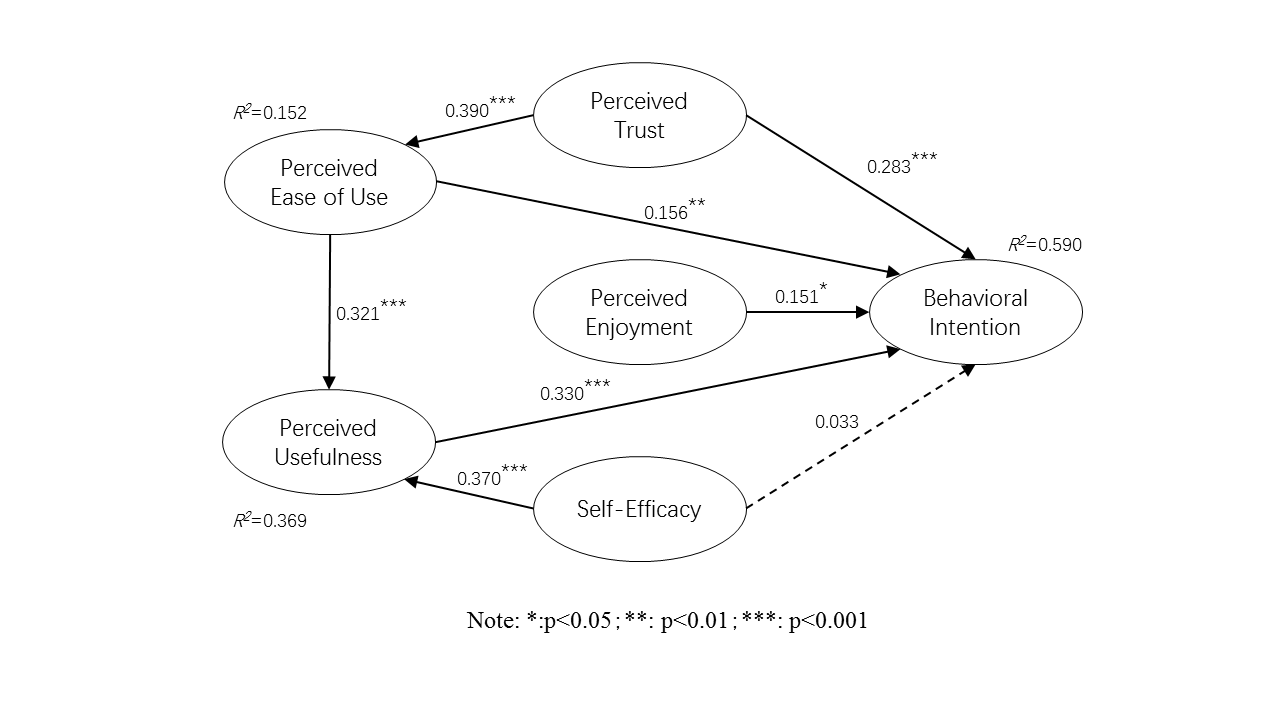

Supplement: S2 Fig — (TIF) [file pone.0256570.s002.tif]
