# Supplementary material for: Research on the use intention of potential designers of unmanned cars based on technology acceptance model
Source: PLoS One. 2021 Aug 20;16(8):e0256570. doi: 10.1371/journal.pone.0256570 (PMC8378682; doi:10.1371/journal.pone.0256570)
Supplement: S1 File — (DOCX) [file pone.0256570.s003.docx]

**SI File. Questionnaire Items**

**Perceived usefulness（PU）**

PU1. Using a driverless car can improve my driving efficiency.

PU2. Using a driverless car can improve my travel efficiency.

PU3. Using a driverless car can reduce my driving stress.

PU4. I will find a driverless car is useful.

**Perceived ease of use（PEOU）**

PEOU1.Learning to operate driverless cars would be easy for me.

PEOU2.I would find it easy to get driverless cars to do what I want to do.

PEOU3.I will find driverless cars easy to use.

**Behavioral intention to use (BI)**

BI1.I intend to use driverless cars in the future.

BI2.I hope to use driverless cars in the future.

BI3.I plan to use driverless cars in the future

**Perceived Trust (PT)**

PT1. Driverless car is dependable.

PT2. Driverless car is reliable.

PT3. Overall, I can trust a driverless car.

**Perceived enjoyment（PE）**

PE1. Using driverless cars would be fun.

PE2. Using driverless cars would be enjoyable.

PE3. Using driverless cars would be pleasant.

**Self-efficacy（SE）**

SE1. If there is a manual, I will be able to use the driverless car.

SE2. If someone tells me how to do it first, I will be able to use a driverless car.

SE3. I think I can use the driverless car without any help.

SE4. I think I can use the driverless car, although I have never used it before.

SE5. If I can find someone to consult, I will be able to use a driverless car.
